# Association Between Satisfaction with Nasal Appearance and Olfactory Function in Patients Undergoing Septorhinoplasty Surgery

**DOI:** 10.34172/aim.2022.51

**Published:** 2022-05-01

**Authors:** Alireza Mohebbi, Amir Yarahmadi

**Affiliations:** ^1^ENT and Head and Neck Research Center and Department, The Five Senses Institute, Hazrat Rasool Hospital, Iran University of Medical Sciences, Tehran, Iran

**Keywords:** Nasal appearance, Olfactory function, Rapid Smell Test, Septorhinoplasty

## Abstract

**Background:**

Any surgery has some complications, and septorhinoplasty is not an exception. The aim of this study was to highlight the relationship between satisfaction with nasal appearance and olfactory function in patients undergoing septorhinoplasty.

**Methods:**

This is a cohort study. In this study, 384 patients aged 18 to 45 years who referred to the Ear, Nose and Throat department at Rasoul Akram hospital and private clinics in 2019 underwent septorhinoplasty. All patients were tested by the Persian Smell Identification Test (PSIT) or Rapid Smell Test (RST) before surgery. They were also reassessed one and three months after surgery. Those patients with dissatisfaction with olfactory function after surgery were also followed up for three months and assessed by PSIT or RST to determine their olfactory dysfunction.

**Results:**

One month after surgery, 73.5% of patients who were not satisfied with their nasal appearance also complained about the olfactory sense. In addition, 1.5% of patients who were satisfied with their nasal appearance also complained about the olfactory sense. There was a significant difference regarding complaints of the olfactory sense between patients satisfied with their nasal appearance and those not satisfied with their appearance (*P*<0.05). Three months after surgery, 78.9% patients who were not pleased with their nasal appearance also had an olfactory complaint. Besides, 0.9% of patients who were pleased with their nasal shape also had an olfactory complaint. There was a significant difference regarding olfactory complaints between patients who were pleased with their nasal shape and those who were not (*P*<0.05).

**Conclusion:**

One and three months after septorhinoplasty, most patients who are satisfied with their nasal appearance have no complaints about their olfactory sense, and most patients who are not satisfied with their nasal appearance complain about the olfactory sense. An appropriate outcome of septorhinoplasty with regard to improving olfactory functional status is accompanied by patients’ satisfaction level of achieving good nasal appearance.

## Introduction

 In today’s society, due to overemphasis on beauty and images in magazines, movies and other media, attention is drawn to appearance, especially in women.^1^ Cosmetic surgery is one of the topics discussed in the sociology of the body.^2^ Shakespeare and Kelly have defined cosmetic surgery as a type of surgery to alter the appearance of the body in the absence of illness, injury or congenital and hereditary malformations that can be a factor in improving quality of life.^3^ The use of surgery and cosmetic procedures has increased dramatically in recent years, such that millions of people volunteer for cosmetic surgeries each year.^4^

 Among all types of cosmetic surgeries, nasal surgery is the most common. In Iran, many people have undergone nasal surgery.^5^ Iran ranks first in the world in the field of rhinoplasty, and a quarter of those who undergo this type of surgery are not satisfied with the surgery and complain about the results.^6^ Respiratory disorders and loss of the sense of smell are the most common complications of nasal cosmetic surgery.^7^ Postoperative deformity is considered a major risk in nasal cosmetic surgery and may cause re-operation in 5%–15% of cases. Revision surgery is needed in approximately 50% of patients.^7^

 The nose is a prominent feature of the face and the most important aesthetic unit. Fracture of the nasal bone is common.^8^ Fracture of the nasal bone due to shots is rare.^9^ Moderate trauma can cause obstruction, discomfort and decline in smell.^9,10^

 Olfactory defects are a prevalent problem. Approximately 5%–15% of patients suffers from anosmia according to reports.^11,12^ Olfactory sense affects several aspects of our life such as quality of life, safety, psychological wellbeing and appetite.^13^

 There may be olfactory defects for patients after rhinoplastic surgery and there is no surprise that surgeons and patients worry about that.^14,15^ Determining a timetable for regaining the sense of smell is a good way to have a mutual relationship with patients so that they can trust their surgeons more^16^ and improve their trust in the aesthetic and/or reconstructive nasal surgery.^17^

 Although there are vast quantities of tests to check the olfactory function, the University of Pennsylvania Smell Identification Test (UPSIT) test is used more frequently by surgeons in the United States^18^; however, this test is invalid in the Iranian population. For this reason, a Persian version, named the Persian Smell Identification Test (PSIT), has been structured and validated and its details have been described previously. The PSIT validity and reliability were measured by Kamrava et al with 99% sensitivity and 81% specificity, with scores of 19 or more indicating normal olfactory function. Also, the authors reported that a score of 17.5 must be the cut-off point for the beginning of olfactory disorders.^19^ Another tool for assessment of olfactory functional status is the Rapid Smell Test (RST) as an instant test for olfactory function which is adopted for the Iranian culture. This test consists of only five highly familiar smells that were chosen from PSIT (coffee, smoke, cinnamon, garlic and banana); consequently, compared with the other tests, it is more comfortable for use in clinics.^20^

 Nowadays, many primary care providers use septorhinoplasty as a procedure of limited clinical benefit.^21^ At the moment, funding is limited and can only be justified on a case-by-case basis. It is therefore vital to establish the value of these processes, before they are to be rejected from National Health Service practice altogether. Only few studies in particular have examined the benefits of patient following septorhinoplasty surgery.^22,23^

 Due to the high incidence of trauma in Iran, fracture of the facial bone and the persistence of about 50% of nasal deformities after trauma, many patients with deformity and tilting noses, cosmetic problems, also suffer from respiratory function problems. The aim of this study was to determine the relationship between satisfaction or dissatisfaction with septorhinoplasty and complaints of olfactory dysfunction.

## Materials and Methods

 This prospective group survey was performed on 384 candidates for septorhinoplasty who referred to a private clinic and the Ear, Nose, and Throat department in Rasoul-e-Akram hospital, Tehran, Iran, from patients with the American Society of Anesthesiologists (ASA) physical status II-III and ages of 18–45 who were candidates for the septorhinoplasty surgery.

 The exclusion criteria were: olfactory defects before surgery, history of chronic rhinosinusitis with or without polyps, history of trauma to the nose, history of previous nasal surgeries, severe allergies, genetic or congenital disorders associated with olfactory disorder, psychological problems, and patients who did not complete the follow-up period.

 All patients underwent general anesthesia and open septorhinoplasty. All patients were tested by the PSIT or RST before surgery, and were reassessed one month and three months after surgery. Those patients who were dissatisfied with their olfactory function after surgery were also followed up for three months and assessed by PSIT or RST to determine their olfactory dysfunction. Regarding the level of satisfaction with surgery, patients who rated operation as “worth it” were considered to be satisfied, and those who rated it as “not worth it” were considered to be dissatisfied. Subjective olfactory complaints were checked before the olfactory identification test. The candidates were asked this question, ‘‘How is your ability in comprehending weak smells?’’ with the response options “no ability” or “normal”. Finally, after performing the relevant tests, the observations and the results of the three measurements were analyzed using statistical methods.

 Data were analyzed with Statistical Package for the Social Sciences version 20.0 (IBM Corp. Released 2011. IBM SPSS Statistics for Windows, Version 20.0. Armonk, NY: IBM Corp). First, to compare PSIT test results and satisfaction level before and after surgery, the McNemar test was used. Also, to evaluated the agreement between the satisfaction levels and PSIT tests of patients undergoing septorhinoplasty before, 1 and 3 months after surgery, the Kappa coefficient test was used. *P* < 0.05 was considered significant.

## Results

 In this survey, 384 patients undergoing septorhinoplasty were admitted to the study. Overall, 36 men and 348 women participated. The mean age of patients was 25.68 ± 5.18 years.

 There was a significant difference in the PSIT test of patients undergoing septorhinoplasty before and 1 month after surgery (*P* < 0.001). There was also an obvious difference in the PSIT test among patients undergoing septorhinoplasty before and 3 months after surgery (*P* < 0.001). However, we found no significant difference in the PSIT test among patients undergoing septorhinoplasty 1 and 3 months after surgery (*P* = 0.063) (Table 1). Three months after surgery, the nasal olfactory levels in 91.4% of patients reached normal (similar to preoperative) levels (Table 1).

**Table 1:** Comparison of PSIT Test in Patients Undergoing Septorhinoplasty between before, 1 and 3 Months after Surgery

**PSIT Test**	**Time of Measurement**		
	**Before Surgery**	**1 Month after Surgery**	**3 Months after Surgery**
Normal, n (%)	384 (100)^*^ (95% CI: 99.0–100%)	343 (89.3)^*^ (95% CI: 85.8–92.2%)	351 (91.4)^*^ (95% CI: 88.1–94.0%)
Abnormal, n (%)	0 (0) (95% CI: 0.00–0.007%)	41 (10.7) (95% CI: 7.7–14.2%)	33 (8.6) (95% CI: 5.9–11.8%)
Odds ratio (95%CI)	0.500 (0.466–0.537)	0.120 (0.084–0.170)	0.094 (0.064–0.138)

PSIT, Persian Smell Identification Test. ^*^ *P* < 0.001

 There was a significant difference in satisfaction with nasal appearance in patients undergoing septorhinoplasty before and 1 month after surgery (*P* < 0.001) as well as before and 3 months after surgery (*P* < 0.001). In this regard, 1 month after surgery, 87.2% (95% CI: 83.5%–90.4%) of patients were satisfied with their nasal appearance, while 3 months after surgery, 90.1% (95% CI: 86.7%–92.9%) of patients were satisfied with the nasal surgery (Table 2).

**Table 2:** Comparing Satisfaction Level with Nasal Appearance in Patients Undergoing Septorhinoplasty between before, 1 and 3 Months after Surgery

**Satisfaction**	**Time of Measurement**		
	**Before Surgery**	**1 Month after Surgery**	**3 Months after Surgery**
Satisfied, n (%)	0 (0%)^*^ (95% CI: 0.0–0.7%)	335 (87.2%)^*^ (95% CI: 83.5–90.4%)	346 (90.1%)^*^ (95% CI: 86.7–92.9%)
Not satisfied, n (%)	384 (100%) (95% CI: 99.0–100%)	49 (12.8%) (95% CI: 9.6–16.5%)	38 (9.9%) (95% CI: 7.1–13.3%)
Odds ratio (95% CI)	0.500 (0.466–0.537)	0.146 (0.105–0.204)	0.110 (0.076–0.158)

^*^ *P* < 0.001

 One month after surgery, 73.5% (95% CI: 68.9%–48.0%) of patients who were not satisfied with their nasal appearance also complained of olfactory sense. Also, 1.5% (95% CI: 0.6%–3.4%) of patients who were satisfied with their nasal appearance also complained of the olfactory sense. There was a significant difference in the complaints of the olfactory sense between patients satisfied and dissatisfied with their nasal appearance (*P* < 0.05). There was a significantly good agreement between the satisfaction level and PSIT test of patients undergoing septorhinoplasty 1 month after surgery (kappa = 0.774, *P* < 0.001). Similarly, after 3 months of follow-up, patients who were not satisfied with the shape of their nose after surgery had a higher rate of complaints of problems such as olfactory disorder, rhinorrhea, nasal congestion, dry skin of the nose, etc. compared to those who were satisfied with the surgery (Table 3). In the results of the PSIT test of all patients after three months, there was a difference in the olfactory functional status between the satisfied and dissatisfied groups.

**Table 3:** Relationship between Olfactory Functional Status and Level of Satisfaction

**Items **	**Satisfied**	**Dissatisfied**	***P*** ** value**
**One Month After**			
Normal olfactory function, n (%)	378 (98.5%) (95%CI: 96.6–99.4%)	101 (26.5%) (95%CI: 21.9–31.1%)	< 0.001
Abnormal olfactory function, n (%)	6 (1.5%) (95%CI: 0.6–3.4%)	283 (73.5%) (95%CI: 68.9–48.0%)	
Odds ratio (95% CI)	0.525 (0.371–0.823)		
**Three Months After**			
Normal olfactory function, n (%)	380 (99.1%) (95% CI: 97.3–99.7%)	81 (21.1%) (95% CI: 17.1–25.5%)	< 0.001
Abnormal olfactory function, n (%)	4 (0.9%) (95% CI: 0.3–2.6%)	303 (78.9%) (95% CI: 74.4–82.8%)	
Odds ratio (95% CI)	0.765 (0.370–0.966)		

## Discussion

 Rhinoplasty is a shared operation between otolaryngology and plastic surgery. Both otolaryngology and plastic surgery studies attempt to reduce invasive ways and create balanced long-term results. Statistics indicate that only about 50% of patients confirm satisfaction as reported by publications, suggesting that acceptable results are not universal. Many patients seek revision.^25^

 In the literature review, no study was found to evaluate the agreement between satisfaction level and the results of PSIT test in patients undergoing septorhinoplasty in short- and mid-term follow-up after surgery. However, many previous studies, similar to the present study, reported improved satisfaction of patients with nasal appearance postoperatively.^25-28^ A study by Baser et al consistent with the findings of the present study, found that functional satisfaction significantly correlates with nasal obstruction in patients undergoing open septorhinoplasty. Also, Nasal Obstruction Symptoms Evaluation scores were found to be decreased significantly after surgery, and Rhinoplasty Outcomes Evaluation (ROE) scores were increased postoperatively.^26^ Consistent with the findings of the present study, Fuller et al found that both nasal blockage and patient satisfaction with their nasal surgery were significantly improved following septorhinoplasty in 2, 4, 6, and 12 months postoperatively.^27^ In a study by Zojaji et al, whose results were similar to ours, it was found that the entire facial ratio altered dramatically after rhinoplasty.^28^ Although patients are more pleased with their nasal shape after surgery, there is no association between patient satisfaction and facial proportions. Studies show that most patients are satisfied with septorhinoplasty.

 According to PSIT examination for non-satisfied patients, there is no remarkable difference between one month and three months after surgery.

 Belli et al reported that those patients who are not satisfied with their nasal shape after rhinoplastic surgery are at greater risk for mental disorders such as depression, anxiety and psychosomatic symptoms.^29^ The advantage of this study is the fact that this was a cohort research. Nonetheless, there were limitations in this study: first, few similar studies were conducted and there were limitations in comparing the findings with other studies. Therefore, it is suggested that similar studies should be designed in future. Second, the sample size was small in the current study. Insignificant findings in the two groups might be due to the small sample size; therefore, it is recommended to recruit larger sample sizes in future studies.

 In conclusion, early after septorhinoplasty, most patients who are satisfied with their nasal appearance have no complaints about their nasal olfactory sense, and most patients who are not satisfied with their nasal appearance complain about their olfactory sense. Appropriate outcome of septorhinoplasty with regard to improving olfactory functional status is accompanied by patients’ high satisfaction level of achieving a good nasal appearance.
